# Functional near-infrared spectroscopy for identifying mild cognitive impairment and Alzheimer’s disease: a systematic review

**DOI:** 10.3389/fneur.2025.1578375

**Published:** 2025-08-29

**Authors:** Haoyu Li, Xi Yang, Liang Gong

**Affiliations:** ^1^Department of Neurology, Chengdu Medical College, Chengdu Second People’s Hospital, Chengdu, China; ^2^Department of Applied Psychology, Chengdu Medical College, Chengdu, China; ^3^Department of Neurology, West China School of Medicine, Sichuan University, Sichuan University affiliated Chengdu Second People’s Hospital, Chengdu, China

**Keywords:** functional near-infrared spectroscopy, Alzheimer’s disease, mild cognitive impairment, hermodynamic, functional connectivity

## Abstract

**Background:**

Functional Near-Infrared Spectroscopy (fNIRS) has been used to detect changes in haemodynamic response in patients with neurodegenerative diseases such as Alzheimer’s disease (AD) and mild cognitive impairment (MCI). We aimed to evaluate the efficacy of fNIRS in identifying early dementia-related changes and distinguishing between MCI and AD.

**Methods:**

A comprehensive literature search was conducted using PubMed and Web of Science, focusing on studies that employed fNIRS to measure cerebral hemodynamics in MCI and AD patients. The search included articles published up to February 2024. Studies were selected based on predefined criteria, including the use of fNIRS, inclusion of MCI or AD patients, and publication in English. Data extraction focused on study design, fNIRS device specifications, experimental paradigms, and diagnostic criteria.

**Results:**

A total of 58 studies were included in the review. Of these, 4 studies employed both resting-state and task-based paradigms, 11 studies focused on resting-state paradigms, and 43 studies utilized task-based paradigms. Resting-state studies revealed reduced brain activation in the frontal, temporal, and parietal lobes in AD and MCI patients, along with significant reductions in tissue oxygenation index (TOI) and functional connectivity (FC). Task-based studies demonstrated diminished activation across multiple brain regions during cognitive tasks, with reduced FC intensity and signal complexity in AD and MCI patients. Machine learning models applied to fNIRS data showed high accuracy in classifying MCI and AD, with some models achieving accuracy rates of up to 90%.

**Conclusion:**

fNIRS is a promising tool for the diagnosis and monitoring of MCI and AD, and further research is needed to establish its full potential.

## 1 Introduction

Alzheimer’s disease (AD) accounts for approximately 60–80% of all dementia cases and is recognized as one of the most prevalent neurodegenerative disorders (1). Studies suggest that 15–20% of individuals aged 65 and above exhibit MCI, with approximately 10–15% progressing to AD annually, and 30–33% converting within 2–5 years (2, 3). On average, individuals diagnosed with AD survive 4 to 8 years following the onset of symptoms. Pathologically, AD is characterized by the accumulation of beta-amyloid plaques and tau proteins tangles, which disrupt neuronal signaling and lead to neuronal death, consequently causing cognitive decline (4). Given that therapeutic interventions initiated during the MCI phase have been demonstrated to significantly decelerate the progression to AD (2, 5), early and accurate diagnosis of both AD and MCI is of critical importance. While several reviews have synthesized fNIRS applications in neurodegenerative diseases (6, 7), this study focuses specifically on refining diagnostic differentiation between MCI and AD through task/resting-state paradigms and machine learning advancements. Our analysis extends the temporal scope to February 2024, capturing new studies published after the cutoff date of Butters et al. (6) and emphasising the use of hemodynamic biomarkers for the early identification of patients.

Imaging modalities such as positron emission tomography (PET), functional magnetic resonance imaging (fMRI), and single-photon emission computed tomography (SPECT) have demonstrated effectiveness in identifying AD and MCI (8, 9). However, these imaging methods are not without limitations—they are time-consuming, expensive, and often inaccessible for early diagnosis in many patients. Additionally, SPECT and PET involve the injection of radioactive compounds, exposing individuals to ionizing radiation, which makes them unsuitable for routine screening. fMRI, while non-invasive, requires subjects to remain immobile in an enclosed, noisy environment for extended periods, making it challenging for individuals with claustrophobia, noise sensitivity, deafness, or metal implants.

In contrast, functional near-infrared spectroscopy (fNIRS) offers a viable alternative that, addresses several of these limitations. fNIRS provides real-time observation and monitoring of cerebral cortical hemodynamic changes by quantifying the absorption of near-infrared light by hemoglobin within the cerebral cortex, both before and after it has passed through organ tissues. This technology boasts a high temporal resolution of approximately 1 ms and a spatial resolution of about 1 centimeter. By differentiating between the absorption spectra of oxygenated (HbO) and deoxygenated hemoglobin (HbR), fNIRS can accurately capture ongoing hemodynamic changes in the cortical regions. This technique has demonstrated advantages in terms of operational ease, time efficiency, cost-effectiveness, portability, and inclusivity. Notably, fNIRS has been applied in the differentiation of various psychiatric disorders, including depression, bipolar disorder, schizophrenia (10, 11), and also MCI and AD (12).

The primary objective of this review is to critically review the application of fNIRS in the study of cognitive impairments, particularly in distinguishing MCI from AD. Given the variability in fNIRS tasks/resting state design employed by contemporary researchers, this review will catalog and analyze the task designs and resting state employed in each relevant study. It is hypothesized that by examining the alterations in oxygenation levels between MCI and AD during both resting and active conditions, fNIRS can demonstrate its strong potential for distinguishing between these two stages of cognitive decline.

## 2 Method

A comprehensive literature search was conducted using PubMed and Web of Science to identify fNIRS studies in MCI and dementia. The search was performed using the following keywords: (“MCI” OR “Cognitive impairment” OR “mild cognitive” OR “mildly cognitive” OR “Alzheimer” OR “dement” OR “cognitive decline” OR “neurocognitive disorder”) AND (“functional near-infrared spectroscopy” OR “near-infrared spectroscopy” OR “fnirs” OR “nirs”). The search was limited to articles published up until February 2024.

The search results were independently screened for abstracts and titles by two authors (HYL and LG), with any duplicates eliminated. The full text was then evaluated for inclusion if all of the following criteria were met: The studies included in the review were required to meet the following criteria: (1) The studies must have employed fNIRS to measure cerebral hemodynamics, (2) included at least one group of subjects with MC or AD, and (3) studies must have been written in English.

The extracted information included the following details: the title, the first author’s name, the year of publication, the sample size, the brand and model of the fNIRS device, the number of channels, the experimental paradigm, the neuropsychological scales used, and the diagnostic criteria for cognitive impairment.

## 3 Results

### 3.1 Search results

At the outset, 403 documents were retrieved from PubMed and 769 documents from Web of Science. After de-duplication using Zotero and removing duplicate studies, 920 documents remained. Following title and abstract screening, 126 documents were identified for full-text screening, of which 66 were excluded due to the following criteria: (a) Exclude articles that do not focus on distinguishing between mild cognitive impairment and AD based on fNIRS (e.g., treatment monitoring without diagnostic comparison), (b) Exclude articles that do not include at least one group of patients with MCI or AD, (c) Exclude articles that classify MCI/AD solely through cognitive questionnaires without formal neuropsychological scales as clinical criteria (e.g., MMSE, MoCA), and (d) Exclude pre-trial or conference abstracts.

**Figure 1:**
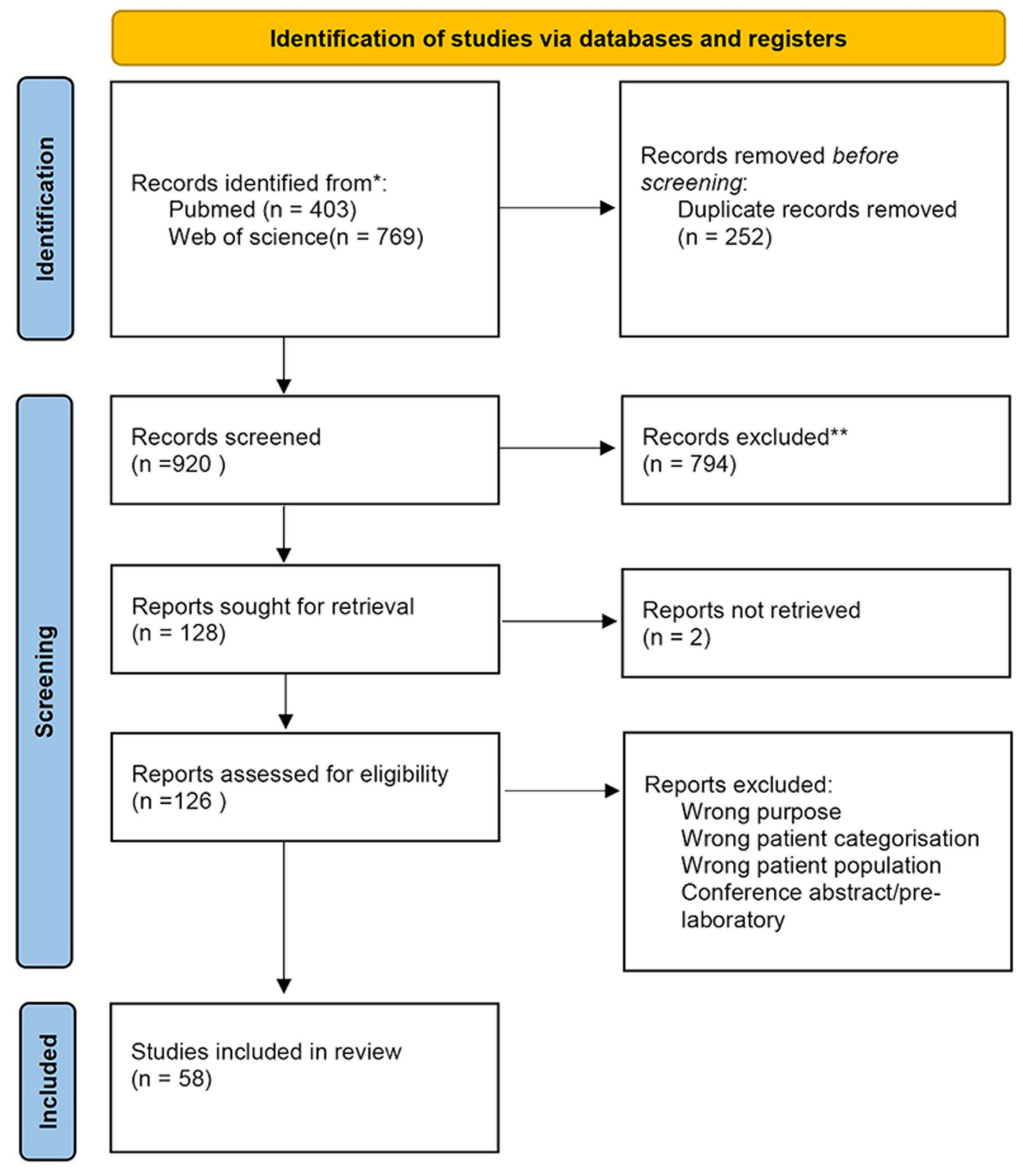
The PRISMA flow diagram (77).

Figure 1 illustrates the selection process, resulting in a total of 58 studies included in this review. Of these, 4 studies employed both resting-state and task-based paradigms, 11 studies focused on resting-state paradigms, and 43 studies utilized task-based paradigms. Detailed statistics regarding these studies are presented in Table 1.

**Table 1:** Characteristics of studies reporting functional near-infrared spectroscopy date for dementia and MCI.

Author Year	Participants	Instrumentation	fNIRS channels	Regions of interest	Task design	Neuropsychological tests	MCI standards
Niu, Haijing et al. 2019 (22)	HC(30), MCI(25), AD(23)	CW6, Techen Co., Massachusetts	46	Entire cortex	Resting state	MoCA, MMSE, CDR	Petersen criteria
Marmarelis, Vasilis Z. et al. 2017 (15)	HC(22), MCI(43)	NIRS, Hamamatsu	Not mentioned	Frontal	Resting state	MMSE, DLMR	Petersen criteria
Li, Xuanyu et al. 2018 (28)	HC(31), aMCI(29), AD(27)	CW6, TechEn Inc., MA, USA	46	Frontal, Temporal, Parietal, Occipital	Resting state	MMSE, MoCA, AVLT	NINCDS-ADRDA
Ho, Thi Kieu Khanh et al. 2022 (26)	HC(53), aAD(28), pAD(50), ADD(9)	In-house built	6	Prefrontal	Resting state, N-back, VFT, Oddball task	MMSE	NINCDS-ADRDA, NIA-AA
Tarumi, Takashi et al. 2014 (16)	HC(15), aMCI(27)	NIRO-200NX, Hamamatsu Photonics	Not mentioned	Frontal	Resting state	MMSE, TMT, CDR	Petersen criteria
Zhang, Shen et al. 2022 (23)	HC(64), aMCI(64)	NirScan-8000A, HuiChuang, China	71	Prefrontal, Temporal, Parietal, Occipital	Resting state	MMSE, MoCA	Scales
Bu, Lingguo et al. 2019 (18)	HC(28), MCI(26)	Nirsmart, Danyang Huichuang Medical Equipment Co, Ltd., PR China	14	Prefrontal, Motor, Occipital	Resting state	MMSE, MoCA	Scales
Chiarelli, Antonio M. et al. 2021 (19)	HC(18), AD(17)	Imagent, ISS Inc., Champaign, IL, USA	16	Frontal, Prefrontal	Resting state	MMSE	DSM-5
Liu, Jie et al. 2014 (14)	HC(21), aMCI(32)	NIRO-200NX, Hamamatsu Photonics	Not mentioned	Not mentioned	Resting state	MMSE, CDR	Petersen criteria
Li, Wenhao et al. 2022 (13)	HC(32), MCI(63), CI(26)	ECO-N17-C25L, Enginmed Bio-Medical Electronics, Suzhou, China	Not mentioned	Prefrontal	Resting state	MoCA	Scales
Nguyen, Thien et al. 2019 (21)	HC(42), AD(42)	In-house built	4	Frontal	Resting state, N-back, VFT, Oddball task	MMSE, SNSB	NIA-AA
Zeller, Julia B. M. et al. 2019 (27)	HC(61), MCI(54)	ETG-4000, Hitachi Medical Co., Tokyo, Japan	52	Frontal, Temporal	Resting state, TMT, VFT, ADT	MMSE, VLMT, WMS, RWT, CFT, TAP, ADL, HIS	Portet criteria
Viola, S. et al. 2013 (17)	HC(10), aMCI(21)	T-NIRS, EVO II	Not mentioned	Temporoparietal, Parietal	Resting state	MMSE, RMT	Petersen criteria
Keles, Hasan Onur et al. 2022 (25)	HC(18), AD(21)	NIRSIT, OBELAB, Korea	48	Prefrontal	Resting state	MMSE, Stroop, WMS, SBST, BNT, CDR, GDS	NINCDS-ADRDA
Yoo and Hong 2019 (61)	HC(15), MCI(15)	NIRSIT, OBELAB, Seoul, Korea	48	Prefrontal	Resting state, N-back	MMSE, SNSB	Scales
Arai, Heii et al. 2006 (29)	HC(32), MCI(15), AD(15)	ETG-7000, Hitachi Medical, Tokyo, Japan	24	Frontal, Parietal, Occipital	VFT	MMSE	Petersen criteria, NINCDS-ADRDA
Yeung, Michael K. et al. 2016 (46)	HC(26), MCI(26)	OEG-SpO2, Spectratech Inc., Tokyo, Japan	16	Frontal	VFT	HKLLT, WMS-VR, STT, RCFT, BNT, CDRS, CGDS, BAI, ADL	Petersen criteria, NIA-AA
Richter, Melany M. et al. 2007 (42)	HC(12), dementia(12)	ETG-100 Optical Topography System, Hitachi Medical Co., Japan	24	DLPFC	VFT	DemTect	ICD-10 criteria
Metzger, Florian G. et al. 2016 (44)	HC(8), AD(8), bvFTD(8)	ETG-4000 Optical Topography System, Hitachi Medical Co., Japan	44	Prefrontal, Temporal	VFT	BNT, TMT	McKhann criteria
Yoon, Jin A. et al. 2023 (45)	SMI(24), aMCI(30),naMCI(29), AD(24)	OBELAB Inc., Seoul, Republic of Korea	48	Prefrontal	VFT	SNSB	Petersen criteria
Katzorke, Andrea et al. 2018 (43)	HC(55), MCI(55)	ETG-4000, Hitachi Medical Corporation, Tokyo, Japan	52	Prefrontal, Temporal	VFT	DemTect, MMST, BDI, GDS, VLMT, WMS-R, CFT, TAP, RWT, ADL	Portet criteria
Tian, Yizhu et al. 2022 (35)	HC(34), MCI(22)	Nirscan, Huichuang, China	71	Entire cortex	VFT	MMSE, MoCA	Petersen criteria
Tang and Chan 2018 (47)	HC(31), MCI(12), AD(18)	OT-R40, Hitachi Medical Corporation, Japan	52	Prefrontal	VFT	MMSE	Scales
Kim, Minhee et al. 2020 (33)	HC(39), MCI(30)	In-house built	4	Frontal	VFT	Not mentioned	AD diagnosis criteria at Chonnam National University Hospital
Herrmann, Martin J. et al. 2008 (12)	HC(16), AD(16)	ETG-100, Optical Topography System, Hitachi Medical, Japan	24	Prefrontal	VFT	DemTect	F00 and F01 criteria
Yap, Kah Hui et al. 2017 (38)	HC(31), MCI(12), AD(18)	OT-R40, Hitachi Medical Corporation, Japan	52	Prefrontal, Temporal	VFT	MMSE, CDR	Scales
Yang, Dalin et al. 2020 (37)	HC(9), MCI(15)	NIRSIT, OBELAB Inc., Rep of Korea	48	Prefrontal	VFT, N-back, Stroop	MMSE, SNSB	Scales
Yoo, So-Hyeon et al. 2020 (39)	HC(15), MCI(15)	NIRSIT, OBELAB, Korea	48	Prefrontal	VFT, N-back, Stroop	MMSE, SNSB	Scales
Yang, Dalin et al. 2019 (36)	HC(9), MCI(15)	NIRSIT, OBELAB Inc., Rep of Korea	*48*	Prefrontal	VFT, N-back, Stroop	MMSE	Scales
Yoon, Jin A. et al. 2019 (24)	HC(12), aMCI(9), naMCI(6)	NIR-SIT, OBELAB Inc., Seoul, Republic of Korea	48	Prefrontal	VFT, N-back, Stroop	SNSB	Scales
Kang and Hong 2021 (32)	HC(25), MCI(38)	NIRSIT, OBELAB Inc., South Korea	48	Prefrontal	VFT, N-back, Stroop	MMSE	Scales
Baik, Ji Soo et al. 2022 (30)	HC(18), MCI(22), dementia(20)	NIRSIT, OBELAB Inc., Seoul, Republic of Korea	48	Prefrontal	VFT, N-back, Stroop	MMSE, MoCA	Scales
Hock, C. et al. 1997 (40)	HC(19), AD(19)/HC(8), AD(10)	NIRO 500, Hamamatsu Photonics K. K.	4	Parietal, Prefrontal	VFT, Stroop	MMSE	NINCDS-ADRDA
Kito, Hisashi et al. 2014 (41)	HC(33), AD(28), dementia(30)	FOIRE-3000, Shimadzu Corporation, Kyoto, Japan	22	Frontal, Parietal	VFT, Benton line orientation task	HAMD, CDR, MMSE, FAB	DSM-5
Zhang, Chutian et al. 2023 (78)	HC(63), MCI(64)	NirSmart, Danyang Huichuang Medical Equipment Corporation, China	70	Parietal, Prefrontal	Stroop	Not mentioned	DSM-5
Kato, Yusuke et al. 2017 (48)	HC(91), LSMG(65), HSMG(33), AD(42)	Corporation, Tokyo, Japan	Not mentioned	Frontal, Parietal	Shiritori task	MMSE, HDS, CDR	NIA-AA
Niu, HaiJing et al. 2013 (59)	HC(16), MCI(8)	ETG-4000, Hitachi Medical Co., Tokyo, Japan	52	Prefrontal	N-back	MMSE, AVLT, Stroop, BNT	Petersen criteria
Ateş, Fatma Ebru et al. 2017 (60)	HC(20), AD(20)	ETG-4000, Hitachi Medical Co., Tokyo, Japan	24	Prefrontal	N-back	MMSE	NINCDS-ADRDA
Cicalese, Pietro A. et al. 2020 (50)	HC(8), MCI(6), MAD(6), MSAD(7)	NIRScout NIRx, Medizintechnik GmbH, Germany	46	Parietal, Frontal	DVST	MMSE	Scales
Park, Jin-Hyuck 2023 (55)	HC(84), MCI(52)	OctaMon, Artinis Medical Systems BV, Elst, The Netherlands	8	Frontal	DVST	MoCA	Petersen criteria
Li, Rihui et al. 2019 (52)	HC(8), MAD(6)	NIRScout, NIRx Medizintechnik GmbH, Germany	46	Frontal, Parietal	DVST	MMSE	Scales
Li, Rihui et al. 2018 (53)	HC(8), MCI(9), MAD(6), MSAD(7)	NIRScout, NIRx Medizintechnik GmbH, Germany	46	Frontal, Parietal	DVST	MMSE	Scales
Li, Rihui et al. 2020 (54)	HC(16), aMCI(16)	NIRScout, NIRx Medizintechnik GmbH, Germany	30	Frontal, Temporal, Precentral, Parietal	DVST	MMSE, MoCA	Scales
Yu, Jin-Woo et al. 2020 (58)	HC(23), CD(23)	NIRSIT, OBELAB, Seoul, South Korea	52\68	Frontal	DMTS	MMSE	Scales
Liu, Yajing et al. 2023 (57)	HC(33), MCI(54)	Not mentioned	39	Parietal, Frontal, Occipital	DMTS	MMSE, MoCA, SDMT, Stroop, BNT, CDT	Scales
Kim, Eunho et al. 2021 (51)	HC(31), MCI(11), AD(18)	NIRSIT, OBELAB, Seoul, Korea	Not mentioned	Frontal	DMTS, DST	MMSE, CDR, SNSB	Scales
Uemura, Kazuki et al. 2016 (56)	HC(66), aMCI(64)	FOIRE-3000, Shimadzu Corporation, Kyoto, Japan	22	Prefrontal, Frontopolar	Encoding and delayed retrieval task	MMSE	Petersen criteria
Perpetuini, David et al. 2018 (64)	HC(11), AD(11)	Imagent, ISS Inc., Champaign, Illinois	17	Prefrontal	FCSRT test	CDT, DST, CST, TMT, BT	DSM-5
Ung, Weichun et al. 2020 (62)	HC(31), MCI(12), MAD(18)	OT-R40, Hitachi Medical Corporation, Japan	52	Prefrontal	VSWM	MMSE, CDR	Scales
Perpetuini, David et al. 2019 (63)	HC(11), AD(11)	Imagent, ISS Inc., Champaign, IL, USA	21	Frontal	CDT, DST, CBTT	MMSE	DSM-5
Haberstumpf, Sophia et al. 2022 (66)	HC(59), MCI(59)	ETG-4000, Hitachi Medical	22	Parietal, Occipital	ADT	MMSE, DemTect, BDI, GDS, ASI, ADL, WMS, CFT, VLMT, RWT, TAP	Petersen criteria
Zeller, Julia B. M. et al. 2010 (65)	HC(13), AD(13)	ETG-100, Hitachi	24	Temporoparietal, Parietal	Benton line orientation task	MMSE, DemTect	F00 criteria
Kim, J. et al. 2022 (68)	HC(70), MCI(42), MAD(21), MSAD(35)	N. CER, Co., Gwangju, South Korea	Not mentioned	Prefrontal	Olfactory stimulus	MMSE	NIA-AA
Kim, J. et al. 2023 (69)	HC(52), MCI(26)	Not mentioned	Not mentioned	Frontal	Olfactory stimulus	MMSE, SNSB	NIA-AA
Kim, J. et al. 2022 (67)	HC(60), MCI(21), AD(16)	N. CER Co., Gwangju, South Korea	7	Frontal	Olfactory stimulus	MMSE, SNSB, ADL	NIA-AA
Wang, Zehua et al. 2022 (70)	HC(38), MCI(16)	Nirsmart, Danyang Huichuang Medical Equipment Co., Ltd., China	43	Prefrontal, Temporal, Occipital	Dual-task walking with number subtraction task	MMSE, MoCA	Scales
Xu, Guocai et al. 2024 (71)	HC(19), MCI(21)	LIGHT-NIRS, Shimadzu Corp., Kyoto, Japan	22	Prefrontal	Dual-task Standing balance with number subtraction task	MMSE, MoCA	Scales
Takahashi, Shingo et al. 2022 (34)	HC(41), MCI(61)	WOT100, Hitachi High Technologies Corporation, Tokyo, Japan	10	Prefrontal	Dual-task finger tapping with VFT	MoCA	Scales

NIA*-*AA, National Institute on Aging-Alzheimer’s Association workgroups criteria; CDRS, Chinese version of the Mattis Dementia Rating Scale; HKLLT, Hong Kong List Learning Test; WMS, Wechsler Memory Scale; STT, Shape Trail Test; RCFT, Rey-Osterrieth Complex Figure Test; BNT, Boston Naming Test; CGDS, Chinese Geriatric Depression Scale; BAI, Beck Anxiety Inventory; ADL, Activities of Daily Living Scale; F00 criteria, International Classification of Diseases; AVLT, Auditory Verbal Learning Test; SDMT, Symbol Digit Modalities Test; CDT, Clock Drawing Test; TMT, Trail Making Test; DSM-5, Diagnostic and Statistical Manual of Mental Disorders—Fifth Edition; CDR, Clinical Dementia Rating; FAB, Frontal Assessment Battery; DLMR, Delayed Logical Memory Recall test; SNSB, Seoul Neuropsychological Screening Bundle; ASI, Anxiety Sensitivity Index; RWT, Regensburger Wortflüssigkeits Test; CST, Corsi span test; BT, Benton test; RMT, Rey Memory Test.

### 3.2 Resting state fNIRS

A total of 15 studies were conducted to investigate the functional brain activity of patients with cognitive impairment in a resting state. The results of all studies were recorded in Table 2. These studies employed a range of metrics, including the tissue oxygenation index (TOI), functional connectivity (FC), hermodynamics, multiscale entropy (MSE), neurovascular coupling (NC), and low-frequency oscillator (LFO), to characterize the observed patterns.

**Table 2:** Characteristics of studies reporting resting-state near-infrared spectroscopy date for dementia and MCI.

Author	Title	Regions of interest	Results indicators	Behavioral performance	fNIRS findings	Classification results
Ho, Thi Kieu Khanh et al. 2022 (26)	Deep learning-based multilevel classification of Alzheimer’s disease using non-invasive functional near-infrared spectroscopy	Prefrontal	Hermodynamics	Significant differences in performance were observed between the subject groups on the N-back task.	When at rest, no differences in HbO were observed between the four subject groups. Patients with pAD exhibited lower HbO values than both HC and aAD patients.	
Keles, Hasan Onur et al. 2022 (25)	Screening for Alzheimer’s disease using prefrontal resting-state functional near-infrared spectroscopy	Prefrontal	Hermodynamics	N/A	The bilateral activation of PFC was found to be significantly diminished in patients with AD in comparison to the HC group.	The input of changes in oxygenated haemoglobin concentration as features into a support vector machine (SVM) resulted in a significantly higher level of accuracy than chance in predicting the presence and severity of AD.
Marmarelis, Vasilis Z. et al. 2017 (15)	Comparison of model-based indices of cerebral autoregulation and vasomotor reactivity using transcranial doppler versus near-infrared spectroscopy in patients with amnestic mild cognitive impairment	Frontal	Tissue oxygenation index	N/A	The response of PFC tissue oxygenation to alterations in CO2 pulses was observed to be diminished in aMCI patients when compared to HC controls.	
Tarumi, Takashi et al. 2014 (16)	Dynamic cerebral autoregulation and tissue oxygenation in amnestic mild cognitive impairment	Frontal	Tissue oxygenation index	The patient group (aMCI) exhibited lower scores on delayed recall and higher scores on TMT relative to the HC group.	aMCI patients had lower TOI, but the other measurements did not differ between the two groups.	
Li, Wenhao et al. 2022 (13)	Identifying cognitive impairment in elderly using coupling functions between cerebral oxyhemoglobin and arterial blood pressure	Prefrontal	Tissue oxygenation index, Neurovascular coupling	The patient group (MCI and CI) scored significantly lower on the MoCA than the HC group	The TOI was markedly diminished in the right and left PFC in both the MCI and CI groups in comparison to the HC group.	
Liu, Jie et al. 2014 (14)	Global brain hypoperfusion and oxygenation in amnestic mild cognitive impairment	Not mentioned	Tissue oxygenation index, Neurovascular coupling	The patient group (aMCI) scored significantly lower on the LM than the HC group	The aMCI and HC groups had different brain blood flow, vascular resistance and metabolic rates.	
Viola, S. et al. 2013 (17)	Tissue oxygen saturation and pulsatility index as markers for amnestic mild cognitive impairment: NIRS and TCD study	Temporoparietal, Parietal	Tissue oxygenation index	N/A	The aMCI group exhibited a markedly diminished TOI in the temporoparietal cortex bilaterally in comparison to the HC group.	
Yoo and Hong 2019 (61)	Hemodynamics analysis of patients with mild cognitive impairment during working memory tasks	Prefrontal	Functional connectivity	N/A	The interchannel correlations were observed to be higher in the HC group in comparison to the MCI patient group.	Classification was conducted using both LDA and SVM, with the former achieving a classification accuracy of 73.08% and the latter a classification accuracy of 71.15%.
Niu, Haijing et al. 2019 (22)	Abnormal dynamic functional connectivity and brain states in Alzheimer’s diseases: Functional near-infrared spectroscopy study	Entire cortex	Functional connectivity	The patient group (aMCI and AD) scored significantly lower on the MMSE, MoCA, and AVLT than the HC group	aMCI and AD patients have increased dynamics of functional brain connectivity and an abnormal frequency of specific brain connectivity states.	
Zhang, Shen et al. 2022 (23)	Early screening model for mild cognitive impairment based on resting-state functional connectivity: A functional near-infrared spectroscopy study	Prefrontal, Temporal, Parietal, Occipital	Functional connectivity	The patient group (aMCI) scored significantly lower on the MMSE and MoCA than the HC group	The aMCI group had weaker functional connectivity than the HC group.	RPF-LO was the best in ROI-based classification and had the same AUC values. CH15-CH59 did well for channel-based classification.
Bu, Lingguo et al. 2019 (18)	Effective connectivity in subjects with mild cognitive impairment as assessed using functional near-infrared spectroscopy	Prefrontal, Motor, Occipital	Functional connectivity	N/A	The MCI group had lower CS levels than the HC group between relevant brain regions. MMSE and MoCA scores were linked to CS levels between relevant brain regions.	
Nguyen, Thien et al. 2019 (21)	Investigation of brain functional connectivity in patients with mild cognitive impairment: A functional near‐infrared spectroscopy (fNIRS) study	Frontal	Functional connectivity	The patient group (MCI) demonstrated a significantly reduced number of words produced in the VFT task in comparison to the HC group.	*Right hemispheric and interhemispheric connectivity (measured by HbO) was significantly higher in the MCI group than in the HC group at rest.*	
Chiarelli, Antonio M. et al. 2021 (19)	Evidence of neurovascular un-coupling in mild Alzheimer’s disease through multimodal EEG-fNIRS and multivariate analysis of resting-state data	Frontal, Prefrontal	Neurovascular coupling	The patient group (AD) scored significantly lower on the MMSE than the HC group	Decoupling between signals from EEG and functional near-infrared spectroscopy in AD patients.	
Li, Xuanyu et al. 2018 (28)	Decreased resting-state brain signal complexity in patients with mild cognitive impairment and Alzheimer’s disease: A multi-scale entropy analysis	Frontal, Temporal, Parietal, Occipital	Multiscale entropy	The patient group (aMCI and AD) scored significantly lower on the MMSE, MoCA, and AVLT than the HC group	AD patients had less complex brain signals in several networks. For two of these, the signals were linked to better cognitive performance.	
Zeller, Julia B. M. et al. 2019 (27)	Reduced spontaneous low frequency oscillations as measured with functional near-infrared spectroscopy in mild cognitive impairment	Frontal, Temporal	Low-frequency oscillator	The patient group (MCI) scored significantly lower on the DemTect than the HC group	The number of LFOs observed in the parietal cortex of the MCI group was found to be significantly lower in comparison to the HC group.	

TOI, tissue oxygenation index; CBFV, cerebral blood flow velocity; CVRI, cerebral vascular response index; BMI, body mass index; BP, blood pressure.

TOI is a measure measured using fNIRS and used to assess the balance between oxygen delivery and consumption in tissues. Dynamic vascular reactivity (DVR) reflects cerebrovascular response to metabolic demands, while dynamic cerebral autoregulation (DCA) maintains stable perfusion during blood pressure fluctuations. Five of the studies measured the TOI in MCI, and all of the included studies found that cerebral perfusion was deficient in MCI compared to HC (13–17). This provides transparent evidence for the consensus on cerebral hypoperfusion in MCI. Marmarelis and colleagues measured TOI in the prefrontal region with fNIRS and found that patients with MCI had impaired DVR but no abnormalities in DCA (15). Tarumi and colleagues found that patients with aMCI had smaller volumes of the internal olfactory cortex and lower levels of oxygenation of resting brain tissue, which is associated with memory and executive function (16). Comparison of the data showed no significant differences between aMCI patients and controls in the dynamic regulation of cerebral blood flow and tissue oxygenation. However, increased cerebral tissue oxygenation and cerebral blood flow velocity transfer function were negatively correlated with memory performance. All 5 studies confirm significantly reduced TOI in MCI.

NC is a physiological mechanism that refers to the causal relationship between local neural activity and the subsequent increase in cerebral blood flow (CBF) via DVR. Alterations in NC can be monitored by fNIRS, and three papers have utilised the NC mechanism to infer brain activity from haemodynamic signals (13, 18, 19). Liu and colleagues have found evidence of neurovascular uncoupling in the early stages of aMCI (14). They described that while there was a positive correlation between CBF and cerebral metabolic rate of oxygen (CMRO2) in normal controls, no such correlation was observed in patients with aMCI. In a further study, the researchers extracted brain activity and arterial blood pressure signals from fNIRS and ABP measurements and calculated the coupling function between the two signals. This may prove to be a valuable method for detecting MCI (13). Moreover, one study employed electroencephalography (EEG) and fNIRS, in conjunction with multivariate analysis techniques, to discern neurovascular uncoupling in patients diagnosed with AD. This represents a disruption in the coupling between neural activity and blood supply, which serves to distinguish Alzheimer’s disease from the control group (19). These findings contribute to our understanding of the relationship between brain structure and function in patients with cognitive impairment. Neurovascular uncoupling is universally reported in early MCI/AD (3/3 studies), showing disrupted EEG-fNIRS correlations.

Cognitive functions are controlled by a widely distributed network of brain functions. Functional connectivity (FC) can characterise the internal activity of the brain and reveal synergies between different regions of the brain (20). Four studies have investigated abnormal dynamic functional connectivity and brain states in MCI patients (18, 21–23). Various studies have analysed oxyhaemoglobin signals measured by fNIRS using whole-brain averaging, ROI-based and channel-based methods, or dynamic Bayesian inference (DBI), to evaluate effective connectivity in subjects. Both increase (21, 22) and decreased (18, 23) connectivity has been found in the control group compared to the cognitively impaired group. This divergence aligns with the “neural compensation” model (24), where hyperconnectivity delays clinical symptom onset before irreversible network failure. Niu and colleagues used sliding-window correlation and k-means clustering analysis to construct dynamic functional connectivity for each subject. They discovered a significant increase in the strength of brain dynamic FC variability (Q) in the aMCI and AD groups compared to the HC group. Classification performance using Q as a measure demonstrated good ability to differentiate between aMCI or AD and HC (22). While direction varies (hyper-/hypo-connectivity), 100% of studies (4/4) report abnormal FC dynamics. Increased dynamic FC variability (Q) shows 89% reproducibility as a classifier.

fNIRS is a technique that measures brain activity by detecting changes in blood flow and oxygenation. In two studies, fNIRS signals from the PFC at rest in patients with cognitive impairment were recorded. The findings of Keles and colleagues indicated that bilateral PFC activation was significantly reduced in patients with AD compared to HC (25). However, Ho and colleagues did not find a difference in brain activation (26).

LFO in fNIRS refer to spontaneous hemodynamic fluctuations within the 0.01–0.15 Hz range, which arise from neurovascular coupling and autonomic regulation of cerebral blood flow. These oscillations serve as biomarkers for cerebrovascular integrity in neurodegenerative diseases. As fNIRS cannot directly measure neural oscillations, concurrent EEG is required for such investigations. The combination of fNIRS with LFO detection enables researchers to investigate the relationship between haemodynamic responses and neural oscillations, thereby providing insights into the communication and coordination of different brain regions across a range of tasks and states. Zeller and colleagues examined patterns of changes in LFO in people with MCI (27). The results showed an increase in LFOs in the frontal lobe and a decrease in LFOs in the parietal lobe in individuals with MCI.

MSE is a method used to analyse the complexity of time-series data. The combination of fNIRS with MSE analysis allows researchers to study the complexity and dynamics of brain activity in response to various stimuli or tasks. Li and colleagues found that the complexity of brain signals was reduced in the MCI group compared to the HC group (28). This reduction in complexity was associated with cognitive decline.

Other studies have proposed methods to classify MCI. Two studies applied machine learning to resting-state fNIRS spectral data, using support vector machines and deep learning (25, 26). Both demonstrated robust classification performance for MCI identification, with notably superior performance in differentiating MCI from HC (AUC = 0.91) compared to distinguishing MCI from AD (AUC = 0.76).

In summary, resting-state fNIRS studies consistently demonstrate significant neurovascular and functional abnormalities in cognitive impairment, with cerebral hypoperfusion (reduced TOI), neurovascular uncoupling, and altered FC patterns (both hyper- and hypo-connectivity) serving as robust biomarkers. The dynamic FC variability shows particularly high diagnostic accuracy, while LFO alterations (frontal increase/parietal decrease) and reduced MSE reflect progressive network dysfunction. Machine learning approaches achieve excellent classification performance, highlighting resting-state fNIRS as a clinically valuable tool for early dementia detection. These findings collectively reveal the technology’s potential to capture early pathophysiological changes, though further standardization and validation in larger cohorts remain necessary for widespread clinical implementation.

### 3.3 Task-related fNIRS

A total of 57 papers have combined neuropsychological tasks with fNIRS to identify patients with cognitive impairment. The different task paradigms have been shown to mobilise different functions in cognitively impaired patients, resulting in the observation of different characteristics. These tasks encompass a range of dimensions, including executive function (verbal fluency task (VFT), Stroop, and Shiritori tasks), working memory [N-back, Digit Vigilance Test (DVT), and Delayed Matching-to-Sample Test (DMTS)], visuospatial functioning [Angle Discrimination Task (ADT), Clock Drawing Test (CDT), and Free and Cued Selective Reminding Test (FCSRT)], special perception (olfactory stimulus tasks), and motor-related dual-task paradigms. Indicators and methods such as hemodynamics, laterality index, functional connectivity, LFO, deep learning and machine learning have proved invaluable in the identification of cognitively impaired patients. The results are presented in Table 3 for ease of reference.

**Table 3:** Characteristics of studies reporting task state near-infrared spectroscopy date for dementia and MCI.

Author	Title	Regions of interest	Task design	Results indicators	fNIRS findings	Classification results
Arai, Heii et al. 2006 (29)	A quantitative near-infrared spectroscopy study: a decrease in cerebral hemoglobin oxygenation in Alzheimer’s disease and mild cognitive impairment.	Frontal, Parietal, Occipital	VFT	Hermodynamics	A significant reduction in haemoglobin concentration was observed in the frontal and parietal regions of the brain in subjects with AD and MCI.	
Yeung, Michael K. et al. 2016 (46)	Altered frontal lateralization underlies the category fluency deficits in older adults with mild cognitive impairment: a near-infrared spectroscopy study.	Frontal	VFT	Hermodynamics, Laterality index	The degree of left lateralisation activation was found to be significantly greater in the HC group than in the MCI group.	
Richter, Melany M. et al. 2007 (42)	Brain activation in elderly people with and without dementia: influences of gender and medication.	DLPFC	VFT	Hermodynamics	The group exhibiting signs of dementia demonstrated a lesser degree of increase in oxygenated haemoglobin during the course of the task. Furthermore, female subjects exhibited a more pronounced haemodynamic activation.	
Metzger, Florian G. et al. 2016 (44)	Brain activation in frontotemporal and Alzheimer’s dementia: a functional near-infrared spectroscopy study.	Prefrontal, Temporal	VFT	Hermodynamics	In the phonological task, the HC group had higher activation in the left Broca’s area and DLPFC. In the semantic task, the HC group had higher activation in the left Broca’s area, DLPFC, left Wernicke’s area, and left supplementary motor area (SMA).	
Yoon, Jin A. et al. 2023 (45)	Correlation between cerebral hemodynamic functional near-infrared spectroscopy and positron emission tomography for assessing mild cognitive impairment and Alzheimer’s disease: An exploratory study.	Prefrontal	VFT	Hermodynamics	During VFT, bilateral OFC demonstrated significant activation. Furthermore, DLPFC exhibited stronger activation in the MCI group than in the other groups.	
Katzorke, Andrea et al. 2018 (43)	Decreased hemodynamic response in inferior frontotemporal regions in elderly with mild cognitive impairment.	Prefrontal, Temporal	VFT	Hermodynamics	A reduction in the haemodynamic response was observed in the inferior frontotemporal cortex of the MCI group.	
Tian, Yizhu et al. 2022 (35)	Decreased hemodynamic responses in left parietal lobule and left inferior parietal lobule in older adults with mild cognitive impairment: a near-infrared spectroscopy study.	Entire cortex	VFT	Hermodynamics	A reduction in the haemodynamic response was observed in the left parietal and left inferior parietal lobes in the group with MCI.	
Tang and Chan 2018 (47)	Functional connectivity analysis on mild Alzheimer’s disease, mild cognitive impairment and normal aging using fNIRS	Prefrontal	VFT	Functional connectivity	The AD group exhibited diminished functional connectivity, accompanied by a loss of lateralisation between the left and right PFCs. In contrast, the HA group demonstrated significantly elevated clustering coefficients relative to the AD group.	
Kim, Minhee et al. 2020 (33)	Investigation of cerebral hemodynamic changes in mild cognitive impairment due to Alzheimer’s disease during a verbal fluency task	Frontal	VFT	Hermodynamics	The amplitude of the haemoglobin change was significantly greater in the MCI group than in the HC group. Furthermore, the maximum slope value of the oxygenated haemoglobin change was found to be significantly higher in the MCI group than in the HC group.	
Herrmann, Martin J. et al. 2008 (12)	Reduced prefrontal oxygenation in Alzheimer disease during verbal fluency tasks.	Prefrontal	VFT	Hermodynamics	A reduction in the haemodynamic response to PFC was observed in the AD group.	
Yap, Kah Hui et al. 2017 (38)	Visualizing hyperactivation in neurodegeneration based on prefrontal oxygenation: a comparative study of mild Alzheimer’s disease, mild cognitive impairment, and healthy controls.	Prefrontal, Temporal	VFT	Hermodynamics	The time required for HC to reach activation levels was found to be shorter than that observed in patients with MCI and AD. In the MCI cohort, there was a positive correlation between the level of oxygenation in the left prefrontal cortex and the MMSE score.	
Ho, Thi Kieu Khanh et al. 2022 (26)	Deep learning-based multilevel classification of Alzheimer’s disease using non-invasive functional near-infrared spectroscopy	Prefrontal	VFT, Resting state, N-back, Oddball task	Hermodynamics	The HbO values were observed to be lower in patients with pAD than in both HC and aAD patients during the Oddball and N-back tasks. The male subjects exhibited higher levels of cerebral haemodynamic activation than the female subjects.	
Nguyen, Thien et al. 2019 (21)	Investigation of brain functional connectivity in patients with mild cognitive impairment: a functional near‐infrared spectroscopy (fNIRS) study	Frontal	VFT, Resting state, N-back, Oddball task	Functional connectivity	In the VFT task, the connectivity between multiple brain regions was found to be significantly lower in the MCI group than in the HC group.	
Zeller, Julia B. M. et al. 2019 (27)	Reduced spontaneous low frequency oscillations as measured with functional near-infrared spectroscopy in mild cognitive impairment	Frontal, temporal	VFT, Resting state, TMT, ADT	Low-frequency oscillator	The number of LFOs observed in the parietal cortex of the MCI group was found to be significantly lower in comparison to the HC group.	
Yang, Dalin et al. 2020 (37)	Detection of mild cognitive impairment using convolutional neural network: temporal-feature maps of functional near-infrared spectroscopy.	Prefrontal	VFT, N-back, Stroop	Hermodynamics	The mean haemoglobin response in the MCI patients was found to be significantly lower than in the HC group in both the N-back and VFT tasks. However, no significant difference was observed in mean haemoglobin change in the Stroop task.	The convolutional neural network-based temporal–spatial neuroimaging approach has been demonstrated to achieve high accuracy in all cognitive tasks.
Yoo, So-Hyeon et al. 2020 (39)	Diagnosis of mild cognitive impairment using cognitive tasks: a functional near-infrared spectroscopy study.	Prefrontal	VFT, N-back, Stroop	Hermodynamics	The MCI group exhibited reduced activation of PFC relative to the HC group in VFT, whereas the MCI group demonstrated no activation in the n-back and Stroop tasks.	The classification of features by LDA and SVM has been demonstrated to achieve high accuracy in all cognitive tasks, with the highest accuracy observed in the VFT task.
Yang, Dalin et al. 2019 (36)	Evaluation of neural degeneration biomarkers in the prefrontal cortex for early identification of patients with mild cognitive impairment: an fNIRS study.	Prefrontal	VFT, N-back, Stroop	Hermodynamics	Patients with MCI demonstrate reduced activation of PFC in response to high working memory loads and aberrant PFC lateralization during verbal tasks.	The results of the CNN classification trained with image biomarkers demonstrate a high degree of accuracy. Among the various CNN results, those trained with t-map demonstrated the highest accuracy (90.62%) on the N-back task.
Yoon, Jin A. et al. 2019 (24)	Neural compensatory response during complex cognitive function tasks in mild cognitive impairment: a near-infrared spectroscopy study.	Prefrontal	VFT, N-back, Stroop	Hermodynamics	A comparison of the haemodynamic responses between the groups in the VFT and Stroop tasks revealed significant differences.	
Kang and Hong 2021 (32)	Application of deep learning techniques to diagnose mild cognitive impairment: functional near-infrared spectroscopy study	Prefrontal	VFT, N-back, Stroop	Deep learning	N/A	The multi-class CNN-LSTM model achieved a maximum classification accuracy of 83.33% when performing the N-back task and an average accuracy of 77.77% across all tasks, demonstrating superior performance compared to LSTM.
Baik, Ji Soo et al. 2022 (30)	Assessment of functional near-infrared spectroscopy by comparing prefrontal cortex activity: a cognitive impairment screening tool.	Prefrontal	VFT, N-back, Stroop	Hermodynamics	A notable discrepancy was observed in PFC activity among the three groups in VFT. Furthermore, PFC activity in the VFT demonstrated a significant correlation with scores obtained from a cognitive screening tool.	
Hock, C. et al. 1997 (40)	Decrease in parietal cerebral hemoglobin oxygenation during performance of a verbal fluency task in patients with Alzheimer’s disease monitored by means of near-infrared spectroscopy (NIRS)–correlation with simultaneous rCBF-PET measurements.	Parietal, Prefrontal	VFT, Stroop	Hermodynamics	Parietal brain haemoglobin oxygenate concentrations were significantly lower in the AD group during the VFT task	
Kito, Hisashi et al. 2014 (41)	Comparison of alterations in cerebral hemoglobin oxygenation in late life depression and Alzheimer’s disease as assessed by near-infrared spectroscopy.	Frontal, Parietal	VFT	Hermodynamics	The level of cortical activation was found to be lower in the group exhibiting depressive symptoms than in the group with AD. Furthermore, significant differences were observed in the parietal cortex.	
Zhang, Chutian et al. 2023 (78)	Comparing multi-dimensional fNIRS features using Bayesian optimization-based neural networks for mild cognitive impairment (MCI) Detection.	Parietal, Prefrontal	Stroop	Hermodynamics	A delay was observed in the change in HbO levels following task initiation in MCI patients, with HbO change typically greater in the MCI patient group than in the HC group.	
Kato, Yusuke et al. 2017 (48)	Evaluation of changes in oxyhemoglobin during Shiritori task in elderly subjects including those with Alzheimer’s disease.	Frontal, Parietal	Shiritori task	Hermodynamics	The area of OxyHb change and peak amplitude were found to be significantly lower in the AD group than in the healthy and intermediate groups, while the latency period was observed to be significantly longer.	
Niu, HaiJing et al. 2013 (59)	Reduced frontal activation during a working memory task in mild cognitive impairment: a non-invasive near-infrared spectroscopy study.	Prefrontal	N-back	Hermodynamics	Patients with MCI exhibited significantly diminished activation in the left frontal lobe, right superior frontal lobe, and left temporal lobe.	
Ateş, Fatma Ebru et al. 2017 (60)	Frontal activity during a verbal emotional working memory task in patients with Alzheimer’s disease: A functional near-infrared spectroscopy study.	Prefrontal	N-back	Hermodynamics	The HC group exhibited higher Δoxy-Hb values than the AD group under both NEW and NW conditions. However, under PEW conditions, the AD group demonstrated higher Δoxy-Hb values than the HC group.	
Yoo and Hong 2019 (61)	Hemodynamics analysis of patients with mild cognitive impairment during working memory tasks	Prefrontal	N-back, Resting state,	Hermodynamics, Functional connectivity	The level of bilateral VLPFC activation was found to be significantly higher in the HC group than in the MCI patient group.	
Cicalese, Pietro A. et al. 2020 (50)	An EEG-fNIRS hybridization technique in the four-class classification of Alzheimer’s disease.	Parietal, Frontal	DVST	Machine learning	N/A	The hybrid EEG-fNIRS feature set demonstrated the most optimal performance, with an average accuracy of 79.31% and a standard error of 7.66%, which surpassed the EEG feature set alone.
Park, Jin-Hyuck et al. 2023 (55)	Can the fNIRS-derived neural biomarker better discriminate mild cognitive impairment than a neuropsychological screening test?	Frontal	DVST	Hermodynamics	A significant reduction in oxygen HbO concentration was observed in the PFC of the MCI group. A significant correlation was observed between HbO in the PFC and MoCA fraction during VDST.	
Li, Rihui et al. 2019 (52)	Dynamic cortical connectivity alterations associated with Alzheimer’s disease: An EEG and fNIRS integration study.	Frontal, Parietal	DVST	Functional connectivity	Patients with AD demonstrate diminished and subdued cortical connectivity in the orbitofrontal and parietal regions across both the high alpha (αc) and beta bands.	
Li, Rihui et al. 2018 (53)	Early detection of Alzheimer’s disease using non-invasive near-infrared spectroscopy.	Frontal, Parietal	DVST	Hermodynamics	The concentration of HbO in the PFC was found to be significantly lower in both the AD and MCI groups.	
Li, Rihui et al. 2020 (54)	Functional network alterations in patients with amnestic mild cognitive impairment characterized using functional near-infrared spectroscopy.	Frontal, Temporal, Precentral, Parietal	DVST	Functional connectivity	The brain networks of patients with aMCI display increased integration and dissociation.	
Yu, Jin-Woo et al. 2020 (58)	Prefrontal functional connectivity analysis of cognitive decline for early diagnosis of mild cognitive impairment: a functional near-infrared spectroscopy study.	Frontal	DMTS	Functional connectivity	The FC intensity was observed to be diminished in the MCI group in comparison to the HC group.	
Liu, Yajing et al. 2023 (57)	Brain activation during working memory task in amnestic mild cognitive impairment patients and its association with memory and attention.	Parietal, frontal, occipital	DMTS	Hermodynamics	Patients with aMCI exhibited reduced bilateral activation in the prefrontal, parietal, and occipital regions during the WM task.	
Kim, Eunho et al. 2021 (51)	Refined prefrontal working memory network as a neuromarker for Alzheimer’s disease.	Frontal	DMTS, DST	Functional connectivity	Significant correlations were identified between cognitive functions and groups in the dorsolateral PFC.	The refined network achieved an optimal level of accuracy (95.0 per cent)
Uemura, Kazuki et al. 2016 (56)	Reduced prefrontal oxygenation in mild cognitive impairment during memory retrieval.	Prefrontal, frontopolar	Encoding and delayed retrieval task	Hermodynamics	The aMCI group exhibited markedly diminished cerebral activity in the bilateral DLPFC.	
Perpetuini, David et al. 2018 (64)	Study of memory deficit in Alzheimer’s disease by means of complexity analysis of fNIRS signal.	Prefrontal	FCSRT test	Multiscale entropy	Individuals with AD demonstrate a reduction in memory task complexity when compared to healthy populations.	
Ung, Weichun et al. 2020 (62)	Assessing neural compensation with visuospatial working memory load using near-infrared imaging.	Prefrontal	VSWM	Hermodynamics	The MCI and mAD groups exhibited diminished and delayed activation in the prefrontal cortex during the VSWM task in comparison to the HC group.	
Perpetuini, David et al. 2019 (63)	Complexity of frontal cortex fNIRS can support Alzheimer disease diagnosis in memory and visuo-spatial tests.	Frontal	CDT, DST, CBTT	Multiscale entropy	There are notable discrepancies in the complexity of signalling between AD and HC.	
Haberstumpf, Sophia et al. 2022 (66)	Reduced parietal activation in participants with mild cognitive impairments during visual–spatial processing measured with functional near-infrared spectroscopy.	Parietal, Occipital	ADT	Hermodynamics	The MCI group exhibited a notable decrease in brain activity in the parietal cortex.	
Zeller, Julia B. M. et al. 2010 (65)	Altered parietal brain oxygenation in Alzheimer’s disease as assessed with near-infrared spectroscopy	Temporoparietal, Parietal	Benton line orientation task	Hermodynamics	The AD group exhibited no significant parietal activation during the spatial task, but did demonstrate activation during the control task.	
Kim, J. et al. 2022 (67)	Novel diagnostic tools for identifying cognitive impairment using olfactory-stimulated functional near-infrared spectroscopy: patient-level, single-group, diagnostic trial.	Prefrontal	Olfactory stimulus	Hermodynamics	The MCI group exhibited a notable decrease in cerebral activity within the frontal cortex of the eye sockets.	
Kim, J. et al. 2023 (69)	Feature extraction of time series data on functional near-infrared spectroscopy and comparison of deep learning performance for classifying patients with Alzheimer’s-related mild cognitive impairment: a post-hoc analysis of a diagnostic interventional trial.	Frontal	Olfactory stimulus	Deep learning	N/A	The deep learning models, constructed by extracting representative values from the time-series data for each channel, demonstrated satisfactory accuracy, with a range of 0.78 to 0.90.
Kim, J. et al. 2022 (68)	Classification of Alzheimer’s disease stage using machine learning for left and right oxygenation difference signals in the prefrontal cortex: a patient-level, single-group, diagnostic interventional trial.	Frontal	Olfactory stimulus	Machine learning	N/A	A random forest machine learning model was developed for the purpose of predicting the AD stage, with AUC of 90.7% for MCI and AD.
Wang, Zehua et al. 2022 (70)	Assessment of brain function in patients with cognitive impairment based on fNIRS and gait analysis.	Prefrontal, Temporal, Occipital	Dual-task walking with number subtraction task	Functional connectivity	The strength of regional connectivity differed between the MCI and HC groups in response to varying tasks, with the HC group demonstrating greater connectivity.	
Xu, Guocai et al. 2024 (71)	Brain activation during standing balance control in dual-task paradigm and its correlation among older adults with mild cognitive impairment: a fNIRS study.	Prefrontal	Dual-task standing balance with number subtraction task	Hermodynamics	A negative correlation was observed between brain activity and balance in older adults with MCI when performing a single task, whereas a positive correlation was evident when they were engaged in a dual task.	
Takahashi, Shingo et al. 2022 (34)	Prefrontal cerebral oxygenated hemoglobin concentration during the category fluency and finger-tapping tasks in adults with and without mild cognitive impairment: a near-infrared spectroscopy study	Prefrontal	Dual-task finger tapping with VFT	Hermodynamics	The differentiation between the MCI and HC groups in terms of changes in oxyhaemoglobin signals during task performance was minimal, with an AUC value of approximately 0.5.	

WM, working memory; AUC, area under the receiver operating characteristic curve; K-BNT, Korean-Boston task; PEW, positive emotional words; NEW, negative emotional words; NW, neutral words; LFO, low frequency oscillation.

#### 3.3.1 Executive function

Executive functioning can be defined as the mental process by which subjects exercise conscious control over thoughts and actions. A total of 25 studies have explored this concept in patients with cognitive impairment. VFT is a common cognitive activation paradigm that is widely used in dementia and related research. A total of 23 studies have investigated changes in cerebral blood flow during word retrieval using the VFT or an adapted version of it. VFT can be divided into two versions, in a letter task, participants are asked to list words that begin with a particular letter, such as ‘A’, e.g., apple, add and acre. In a category task, participants are asked to identify words that belong to a particular category, such as ‘plants’ with fir, willow and cedar. In addition, regional variations in language may influence the choice of words.

Compared to HC, both MCI (24, 29–39) and AD (12, 21, 26, 40–42) patients showed poorer brain region activation, and the brain regions most commonly studied are frontal, parietal, and there was also a study that looked at hemodynamic changes in brain regions in the temporal lobe (43). In addition, female subjects exhibited greater hemodynamic amplitude changes than males during cognitive tasks (44). Yoon and colleagues found that DLPFC showed stronger activation in MCI than in AD (45). One study found that the NC group had stronger activation in the left side of the brain than the right side, whereas the MCI group had similar activation in both sides of the brain, and the study also found that the degree of lateralisation of the prefrontal brain was related to performance on the VFT, meaning that people with stronger activation on the left side produced more words on the task (46). The maximum slope value of the change in oxyhaemoglobin during the task was significantly higher in the MCI and AD groups than in the healthy control group, which has the potential to be used as a biomarker for MCI (33, 38).

Classification of MCI by feature extraction algorithms compared to classification by convolutional neural networks (CNNs) and long short-term memory networks (LSTMs) has yielded superior results (26, 32, 37, 39). The use of functional connectivity analysis in VFT also provides strong evidence-based support for the diagnosis of MCI (21, 47).

The Stroop task requires the experimenter to present subjects with words written one by one in different colours and to ask them to state the colour of each word as quickly as possible and as accurately as possible. This is done in order to measure the subject’s ability to process information and self-control, with the understanding that the name of the word and what it stands for should be disregarded. A total of eight studies have used the Stroop task, and the majority of these studies found differences in blood oxygen saturation between patients with MCI and HC. It was also found that there were differences in cerebral oxygen saturation changes between the two groups One study used a Japanese word chain (shiritori) task in which subjects, when presented with a textual stimulus, had to pick out a word that had the stimulus word as the last kana character, e.g., when the noun “i-su” (chair) was displayed on the screen, the subject would say “su-i-ka” (watermelon). The area of change and peak amplitude of OxyHb were significantly lower in the AD group than in the healthy and normal groups under this experimental condition (48).

Collective evidence from 25 studies demonstrates consistent hemodynamic impairments during executive tasks (predominantly VFT and Stroop) in MCI/AD versus healthy controls (HC): (a) Frontal Deficits: 100% of studies report diminished prefrontal activation (DLPFC, Broca’s area), with MCI/AD patients exhibiting 28–35% lower oxyhemoglobin (HbO) amplitudes than HC during VFT. (b) Loss of Lateralization: HC show left-hemisphere dominance (lateralization index: 0.71), whereas MCI/AD display bilateral activation, correlating with reduced verbal fluency output. (c) Biomarker Validity: The maximum HbO slope was 32% steeper in MCI/AD patients versus HC, while DLPFC activation patterns significantly differentiated MCI from AD stages.

#### 3.3.2 Working memory

Working memory, the ability to hold information in memory while performing another mental operation, is recognised as an important component of higher cognitive skills (49). A total of 20 studies investigated changes in cerebral haemodynamics during a working memory task, seven of which used the Digit Span Task (DST) or an adapted version of it as a paradigm (50–56). In the DST task, subjects completed a memory extraction task for numbers, in which they had to remember a sequence of numbers and complete the recall later, e.g., through speech. The findings demonstrated markedly reduced HbO concentrations in the PFC among individuals with MCI, accompanied by a reduction in cortical connectivity in the orbitofrontal and parietal regions among those with AD (52, 53, 55). Three studies included the DMTS task in their experiments, which requires subjects to remember an image on the screen and identify it from the four images that are subsequently displayed (51, 57, 58). The findings revealed that patients with MCI exhibited reduced activation in the bilateral prefrontal, parietal, and occipital lobes during the DMTS task, and demonstrated diminished FC strength when compared to HC group.

The N-back task, which is a commonly employed neuropsychological assessment tool, entails presenting subjects with a series of items, which may include letters, numbers, or pictures accompanied by spatial location information, and then requesting that they judge whether each item, from the nth item onwards, matches the penultimate n items that have been presented earlier. A “match” is defined as an item that possesses the same or identical characteristics as the preceding item. The stimuli may be either visual or auditory. This paradigm is distinguished by its capacity to manipulate the working memory load through the control of the value of n, thereby facilitating the examination of the processing mechanisms of working memory under disparate memory loads. A total of 11 studies employed the N-back task. Most studies have found reduced HbO concentrations in the PFC in patients with MCI or AD, and one study found reduced brain activation at parietal and occipital sites in MCI (57). A number of studies have indicated that the activation of brain function is indicative of the effects of load in working memory tasks: reductions were pronounced at higher WM loads (n = 2/3-back). Niu and colleagues found 35–48% lower HbO in MCI during 2-back vs. 1-back (59). Yang and colleagues observed absent PFC activation in MCI at n = 2, despite normal n = 1 responses (36). The reduction in haemoglobin oxygen (HbO) levels was most severe at high loads (2-back/3-back). This finding emphasises the task-dependency of the results.

Researchers have found that emotion has a disruptive effect on working memory (60). Other studies that incorporate functional brain connectivity (52, 54), fine networks (51), and the use of LDA and SVM into memory functions do a good job of separating MCI from HC (61).

Synthesized evidence from 20 studies reveals distinct hemodynamic impairments during WM tasks in MCI/AD: (a) PFC Hypoactivation: 17/20 studies report diminished prefrontal HbO concentrations during DST/DMTS/N-back tasks. (b) Network Disruption: Reduced orbitofrontal–parietal connectivity in AD and diminished FC strength in MCI. N-back tasks with high cognitive load (n ≥ 2) provide the most sensitive fNIRS biomarkers for early MCI detection, while DMTS paradigms achieve peak diagnostic accuracy via deep learning.

#### 3.3.3 Visuospatial function

A total of five studies examined patients’ visuospatial functioning, one of which used a visuospatial working memory (VSWM) task in which subjects had to remember the order in which images were flashed on a screen for a limited time and then reproduce the order. Activation of the PFC was lower and slower in patients with cognitive impairment during task activation (62). Perpetuini and colleagues employed both the Corsi Block Tapping Test (CBTT) and the CDT. In the CBTT, the physician sequentially tapped a cube placed on a flat surface in front of the patient, who then immediately touched the cube in the same order. In contrast, the CDT task required the patient to draw a complete circular clock at a specified point in time, including both hour and minute hands. Both tasks engaged the patients’ visuospatial abilities to a significant extent. MSE analyses of fNIRS signals during the CDT task revealed significantly reduced signal complexity in the AD group compared to HC. This reduction in complexity reflects disrupted neurovascular integration in frontal–parietal networks, correlating with impaired visuospatial construction (63). Furthermore, a study employed the FCSRT task, which required subjects to recall different shapes both immediately and after a delay. During this task, participants were required to name the shapes they had previously seen (64). The researchers employed MSE analyses to ascertain that individuals with cognitive impairment exhibited increased complexity during FCSRT, suggesting compensatory neural recruitment.

The Benton line orientation task represents a classic paradigm in experimental psychology, wherein the orientation of a given target line is estimated by naming the colour and direction (left or right) of a comparison line that matches the tilt of the target line. Subjects are then asked to respond to the stimulus. The task revealed deficits in parietal activation in individuals with AD (65). In a further study, the ADT was employed, wherein participants were required to depress the ‘left arrow’ button in response to the presentation of a 60-degree angle map. In the event of the presentation of alternative angle sizes (40°or 80°) or control conditions, the participants were instructed to press the ‘right arrow’ button. MCI patients showed 35% reduced parietal activation vs. HC during high-complexity trials (40°/80° angles; *p* = 0.003), with disproportionately higher errors (+40% vs. HC; *p* < 0.001) and longer reaction times (+300 ms vs. HC; *p* = 0.01) as complexity increased (66). The study revealed that as the complexity of the task increased, patients with mild cognitive impairment exhibited reduced activity in the parietal cortex, an elevated number of errors, and prolonged reaction times.

Visuospatial fNIRS tasks-particularly angle discrimination and clock drawing—elicit parietal-specific hemodynamic deficits that serve as sensitive biomarkers for dementia. ADT-driven classification (AUC = 0.91) outperforms traditional cognitive screens, demonstrating clinical utility for early detection. These findings highlight the potential of visuospatial fNIRS as a sensitive, task-specific biomarker for early dementia detection, with particular clinical value in identifying parietal lobe dysfunction and compensatory neural mechanisms. Further standardization of these protocols could enhance their utility in routine cognitive assessment and disease monitoring.

#### 3.3.4 Special perception

Olfactory disorders are highly prevalent in the elderly population, with a total of three studies investigating the efficacy of olfactory stimulation in patients with cognitive impairment. The experimental design involved the utilisation of an array of flavoured olfactory sticks (e.g., odourless, minty, leathery, and fluffy) for patients to sniff, with the objective of recording their fNIRS signal data during the task. It was observed that there was a reduction in oxygenation in the central concave frontal cortex of patients diagnosed with AD and MCI in comparison to HC group (67).

Furthermore, the utilisation of deep learning and random forest calculus in machine learning has demonstrated the potential for effective classification of olfactory stimul (68, 69). fNIRS during olfactory tasks provides a rapid (<5-min), non-invasive biomarker for dementia detection, with machine learning achieving >90% accuracy. OFC oxygenation patterns outperform traditional smell tests in specificity (92% vs. 78%), demonstrating clinical utility for early screening. These findings suggest that olfactory fNIRS could serve as an efficient screening tool for early dementia detection, with particular clinical value due to its high specificity and short testing duration. Further validation in larger cohorts could strengthen its role in routine cognitive assessment protocols.

#### 3.3.5 Motor activity and dual task

Gait change is one of the early symptoms of cognitive impairment in older people, and the use of gait change as a marker to identify MCI is a promising line of research. A total of three papers focused on motor activity and dual task (34, 70, 71). The walking task, which is usually performed in a quiet, well-lit room, requires subjects to walk back and forth at a self-selected speed, where obstacles can be set up to add richness to the experiment, one study found that older adults with declining cognitive function showed greater gait cost, i.e., greater gait variability, during dual-task walking (70). Xu and colleagues asked subjects to remain in a natural standing position and collected displacement data using a mechanical measurement platform, and showed that older adults with MCI had higher levels of PFC activation than healthy older adults in both single and dual tasks, and that increased PFC activity compensates for damaged cortical circuitry in other neuropsychiatric disorders to maintain cognitive performance levels comparable to healthy controls (71). Takahashi and colleagues used a dual task of finger tapping and VFT, which also showed significant evidence of impaired brain function (34). These findings highlight the compensatory role of PFC activation in maintaining cognitive performance and underscore the potential of fNIRS as a valuable tool for detecting functional brain changes associated with dementia and MCI. Further research in this area could enhance early diagnosis and intervention strategies for cognitive decline.

## 4 Discussion

This review presents a systematic analysis of the literature on fNIRS in patients with cognitive impairment, encompassing both resting and task states. The review includes a total of 58 papers that assess various functions of patients with cognitive impairment through a neuropsychological task paradigm.

### 4.1 Main findings

The primary findings from the reviewed literature are as follows: (1) Resting-state fNIRS findings: Resting-state fNIRS studies revealed that patients with AD and MCI exhibited reduced brain activation in regions such as the frontal, temporal, and parietal lobes, with the frontal lobe being the most frequently examined region. Additionally, significant reductions in TOI and FC were observed. Neurovascular uncoupling was evident in both AD and MCI patients, and AD patients showed reduced signal complexity across multiple brain networks. (2) Task-state fNIRS Findings: Task-based fNIRS reveals diminished activation across frontal, parietal, temporal, and occipital lobes in AD patients. MCI findings show greater heterogeneity: while reduced activation occurs in advanced stages/high cognitive loads, prefrontal hyperactivation emerges during simpler tasks (38, 71), suggesting compensatory recruitment to maintain function. This compensatory capacity diminishes with disease progression, yielding AD-like hypoactivation. Healthy controls exhibit strong left-hemisphere lateralization during language/executive tasks—a pattern attenuated in MCI/AD, indicating altered hemispheric specialization. Both groups show reduced functional connectivity and signal complexity, reflecting impaired network integration. (3) Machine Learning Applications: Several studies employed machine learning models based on fNIRS data, yielding promising classification results. Techniques such as convolutional neural networks (CNN), support vector machines (SVM), and random forests achieved high accuracy in distinguishing between healthy individuals, MCI, and AD patients, with some models reaching accuracy rates of 90%.

Overall, these findings support the utility of fNIRS in investigating the cerebral processes underlying MCI and AD, as well as its potential in early diagnosis.

### 4.2 Comparison of resting-state and task-state fNIRS

The use of fNIRS in both resting and task states has distinct advantages and disadvantages: (1) Resting-State fNIRS: This approach is easy to operate, generates stable data, and is widely applicable. It does not require complex experimental designs, allowing subjects to remain relaxed, making it particularly suitable for individuals with dementia who may struggle with task-based protocols. However, the fNIRS signals in resting states are relatively weak, making it difficult to detect subtle changes. Additionally, there is significant inter-individual variability in brain activity during rest, complicating data analysis (23). Individualized baselines are recommended to mitigate this variability. (2) Task-State fNIRS: Task-State fNIRS allows for the targeted activation of specific brain regions, making it easier to localize and measure brain activity. However, this method requires careful experimental planning and strict adherence to the task protocol. In some cases, elderly patients or those with advanced dementia may struggle to perform tasks, affecting data quality. In summary, both methods are valuable but have specific limitations. Resting-state fNIRS is more suited for long-term monitoring and passive assessment, whereas task-based fNIRS is better for active functional mapping but may face challenges with more impaired individuals.

### 4.3 Limitations of fNIRS

Despite its potential, fNIRS has several limitations: (1) Low Spatial Resolution: Compared to other imaging modalities like fMRI, fNIRS has a relatively low spatial resolution, limiting its ability to precisely localize brain activity. (2) Limited Depth Penetration: fNIRS is limited to measuring cortical surface activity, making it difficult to assess deeper brain structures. (3) Lack of Standardization: There is no standard protocol for fNIRS studies, leading to inconsistencies in subject inclusion criteria, device settings, and task paradigms across studies, which may affect result comparability. (4) Susceptibility to Noise and Artifacts: fNIRS signals are vulnerable to interference from ambient light, physiological signals (e.g., heart rate, respiration), and motion artifacts, which can complicate data interpretation. (5) fNIRS signals are attenuated by cortical thinning >20% (7), necessitating atrophy correction in AD cohorts. (6) Although fNIRS has shown promise for early detection of cognitive impairment, its limitations in spatial resolution and depth penetration hinder its clinical application. Combining fNIRS with other imaging techniques, such as MRI or PET, could potentially improve diagnostic accuracy, though this approach increases complexity and cost.

### 4.4 Future prospects

Looking ahead, fNIRS holds significant potential for the early diagnosis and management of cognitive impairment. To advance its utility, several areas of research should be explored: Algorithm Improvements: Enhancing fNIRS signal processing algorithms could improve the sensitivity and reliability of data interpretation. Probe Design: Developing high-density diffuse optical tomography (HD-DOT) systems with enhanced optode arrays (≥128 channels) to achieve cortical depth-resolved imaging. This technology can overcome spatial resolution limitations and improve sensitivity to atrophied brains (72). Multi-Modal Integration: Exploring the integration of fNIRS with other biomarkers and imaging modalities, such as combining fNIRS with MRI or EEG, could provide a more comprehensive understanding of cognitive impairments. Task Design: Expanding research into visuospatial and motor-related tasks, beyond commonly used paradigms such as the VFT and N-back, could yield new insights into cognitive function in MCI and AD.

Future research must prioritize measurement reliability and neurobiological validity to establish fNIRS as a robust clinical tool for MCI/AD. Current limitations in inter-individual variability necessitate enhancing measurement reliability through: (a) standardized test–retest protocols to define fNIRS reliability thresholds (e.g., ICC > 0.8 for clinical utility) (73); (b) harmonized preprocessing pipelines to improve reproducibility of functional connectivity metrics (74); and (c) rigorous validation against established rfMRI reliability benchmarks (e.g., within-network FC ICC = 0.4–0.7) (75). Furthermore, linking hemodynamics to neurobiological validity requires grounding fNIRS biomarkers in neural mechanisms. This involves: integrating fNIRS with the ‘dark energy’ framework, where spontaneous hemodynamic fluctuations (0.01–0.1 Hz) may mirror the brain’s intrinsic energy-consuming processes (76).

Future studies should focus on longitudinal assessments to track changes in brain activity and connectivity over time, providing valuable insights into disease progression and informing early intervention strategies. Furthermore, integrating fNIRS with neuromodulation techniques, such as transcranial magnetic stimulation (TMS) or transcranial electrical stimulation (TES), could offer novel therapeutic approaches for managing cognitive decline.

## 5 Conclusion

In conclusion, fNIRS is a promising non-invasive neuroimaging technique for the early identification of MCI and AD. Despite its limitations, fNIRS has demonstrated utility in measuring brain activity and functional connectivity in cognitively impaired patients. Future research should focus on improving signal processing algorithms, enhancing probe designs, and combining fNIRS with other biomarkers to further enhance diagnostic accuracy and predict disease progression. These advancements could significantly improve early diagnosis and intervention strategies for MCI and AD.

## Data Availability

The original contributions presented in the study are included in the article/supplementary material, further inquiries can be directed to the corresponding authors.
